# Medical students experience in working in a public COVID-19 telehealth program: a descriptive study

**DOI:** 10.1186/s12909-024-05722-6

**Published:** 2024-07-12

**Authors:** Thais Marques Pedroso, Isabela Muzzi Vasconcelos, Caroline Lopes de Amorim, Laryssa Reis Coelho, Maria Augusta Matos Corrêa, Virgílio Barroso de Aguiar, Mayara Santos Mendes, Leonardo Roever, Clara Rodrigues Alves de Oliveira, Milena Soriano Marcolino

**Affiliations:** 1 School of Medicine, Universidade Federal de Minas Gerais, Avenida Professor Alfredo Balena 190, Belo Horizonte, Brazil; 2 Mucuri School of Medicine, Universidade Federal dos Vales do Jequitinhonha e Mucuri, Rua Cruzeiro, 01, Teófilo Otoni, Brazil; 3 Telehealth Center, University Hospital, Universidade Federal de Minas Gerais, Avenida Professor Alfredo Balena 110, Belo Horizonte, Brazil; 4 Department of Clinical Research, Brazilian Evidence-Based Health Network, Uberlândia, Brazil; and Gilbert and Rose - Marie Chagoury School of Medicine, Lebanese American University, Beirut, Lebanon; 5 Telehealth Center, University Hospital, and Telehealth Network of Minas Gerais, Universidade Federal de Minas Gerais, Avenida Professor Alfredo Balena 110, Belo Horizonte, Brazil; 6 Department of Internal Medicine, Medical School and Telehealth Center, Institute for Health Technology Assessment, Rua Ramiro Barcelos, 2350, building 21 - 507, Porto Alegre, Brazil

**Keywords:** COVID-19, Students, medical, Surveys and questionnaires, Telemedicine, Telemonitoring

## Abstract

**Background:**

Given the health and social needs generated by the COVID-19 pandemic, the Telehealth Network of Minas Gerais, Brazil, implemented a teleconsultation and telemonitoring program to assist patients with suspected or confirmed COVID-19, the TeleCOVID-MG program. The telemonitoring service was conducted by medical students, under the supervision of a physician. The main goal of this study was to analyze the experience of the students while collaborating on the aforementioned telemonitoring program.

**Methods:**

A questionnaire with 27 questions was developed to address the participation of the students in the telehealth program. The questionnaire included questions about the student’s profile, the system usability, and the satisfaction in participating in such a telehealth program. The questionnaire was generated on Google Forms® platform and sent via email to each student who was part of the telemonitoring team.

**Results:**

Sixty students were included in the analysis (median age 25 years-old [interquartile range 24–26], 70% women). Of those, 61.6% collaborated on the telehealth program for more than 6 months, 65.1% performed more than 100 telemonitoring calls, 95.2% reported difficulties in contacting the patient through phone calls; 60.3% believe some patients might have felt insecure about being approached by medical students and not by graduate professionals; and 39.6% reported eventual system instabilities. The main strengths reported by the students were related to the system usability and to the self-perception of the quality of healthcare delivered to the patients. Even though 68.3% of the students mentioned technical difficulties, 96.6% reported that they were promptly solved. Finally, 98.3% believed that the program was useful and would recommend it to an acquaintance.

**Conclusion:**

This study reports a successful experience of undergraduate medical students in a COVID-19 telemonitoring program. Overall, the medical students were satisfied with their participation, especially considering the continuity of clinical practice remotely during a period of classes suspension during the COVID-19 pandemic and their important role in the assistance of patients from low-income regions, which has minimized the health system burden in an emergency context.

**Supplementary Information:**

The online version contains supplementary material available at 10.1186/s12909-024-05722-6.

## Background

From the beginning of 2020, due to the COVID-19 pandemic, the world faced an unprecedented emergency regarding the provision of healthcare [1, 2]. COVID-19 not only challenged, but also enabled a series of changes in patient care, including the boom of the use of information and communication technologies on a large scale [3]. The COVID-19 pandemic was a potent booster for the development and spread of telehealth services worldwide [4, 5]. The number of teleconsultations skyrocketed, playing an essential role in maintaining access to care and reducing the virus spread by allowing remote health consultations in different fields, which contributed to minimizing the overcrowding in emergency care units and hospitals [6].

The use of information and communication technologies was also essential for the adaptation of the academic activities, enabling remote teaching in the schools and universities which had face-to-face classes suspended until vaccines were available for COVID-19 [7]. In some instances, the students provided support to the telehealth activities. However, data is scarce about their experience in this field.

Therefore, the context of COVID-19 pandemic resulted in a great paradigm shift in delivering healthcare and information. At the same time, there is scarce data evaluating medical students’ experiences in this field, which is crucial to strengthen the strategies that aim to implement the use of information and communication technologies beyond emergency periods. Thus, this study aims to analyze and discuss the student’s perceptions about collaborating in a public COVID-19 telehealth program.

## Methods

### Study design

This is a descriptive study, carried out between August and November, 2022. It had a convenience sample, in which it was intended to include all undergraduate medical students who participated in the telemonitoring. The study was approved by the Brazilian National Commission for Research Ethics (CAAE 35953620.9.0000.5149).

### The TeleCOVID-MG service

The TeleCOVID-MG is a public telehealth program developed by the Telehealth Network of Minas Gerais (TNMG) to assist patients with suspected or confirmed COVID-19. TNMG is a large public telehealth service formed by a partnership between seven public Universities of Minas Gerais, in the Southeast region of Brazil. It develops different activities in assistance, education, and research fields [8, 9].

The TeleCOVID-MG has three main goals, as described elsewhere: (i) assessing and managing patients with flu-like symptoms, (ii) monitoring patients with COVID-19 (iii) providing the general population with updated information about COVID-19 [10].

The program works through the intersection of four different levels: the user’s gateway to the program is made through a chatbot or a phone call, that represents the level 1; level 2 represents the nursing staff; level 3 represents the medical staff; and level 4 represents the telemonitoring service, composed mainly by medical students [10].

**Fig. 1:**
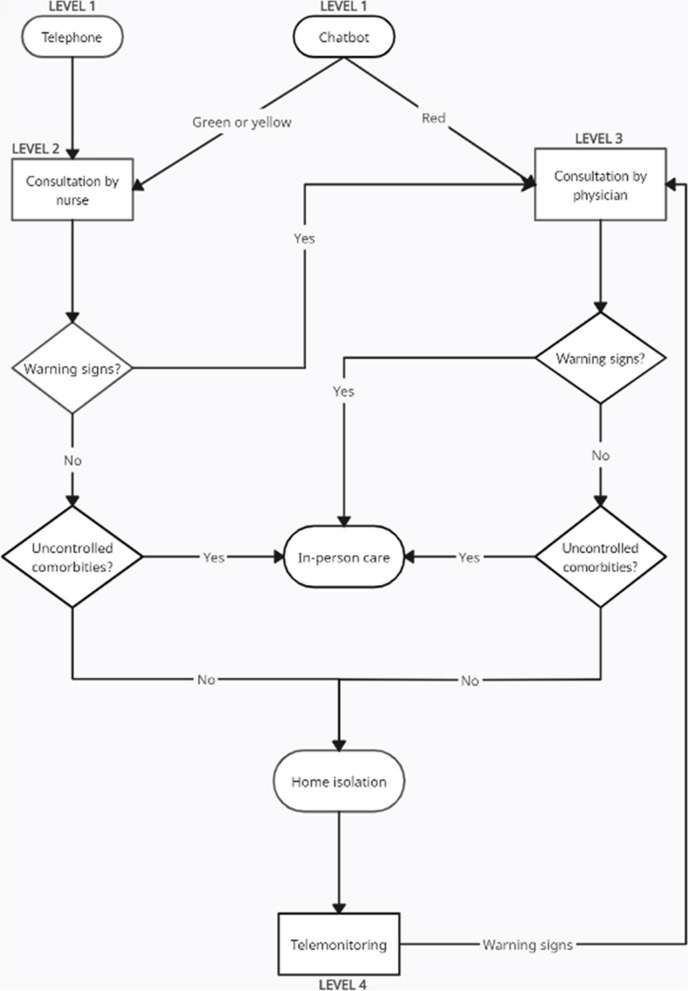
TeleCOVID-MG service workflow. The red, yellow, and green triage rating corresponds to the risk of complications from COVID-19. The green one corresponds to patients with mild to moderate symptoms without other health conditions. As for the yellow one, it corresponds to patients with comorbidities, such as systemic arterial hypertension and diabetes mellitus. For the red triage rating, patients would have warning signs: dyspnea, hypotension and refractory fever (when there is a febrile condition for 3 days or that disappeared after 2 days and returned later)

The patient screening is performed at level 1, based on a scale of colors: green for patients with mild to moderate symptoms without other health conditions; yellow for patients with comorbidities; and red for patients with warning signs (dyspnea, hypotension and refractory fever). The triage rating is detailed on Fig. 1. The patient teleconsultation is performed by nursing or medical staff, according to the severity of their symptoms, at the level 2 or 3. The patient telemonitoring is performed at the level 4, which is composed of undergraduate medical students, who were affiliated to the Telehealth Center or TNMG through extension or scientific initiation programs, and their contributions to the teleassistance was part of their responsibilities within their affiliated projects. The enrolment process was composed of curriculum analysis and interview, and the students could be affiliated voluntarily or through scholarship.

The telehealth service worked in an integrated manner with local in-person services through agreements with local managers, ensuring that it was not a parallel service but a fully integrated one.

The undergraduate students were working under the supervision of medical professors with expertise in telemedicine and primary care and with specialties in Internal Medicine, Family and Community Medicine and Infectious Diseases, which were contracted specifically for the roles. This supervision was done through regular group video calls, and constant communication through messages app (Whatsapp®) and calls. The students received a certificate of participation at the end of their contribution time, which could be used as mandatory extension hours for graduation or could count points to national and regional residency programs enrollments [10]. The students were not evaluated for learning practices through the experience, as their contribution was not part of their formal graduation programs.

TeleCOVID-MG was first implemented in two medium-sized Brazilian cities, Teófilo Otoni e Divinópolis, where the program was kept in operation from May 2020 to December 2021. These cities have public universities that integrate the TNMG, together with *Universidade Federal de Minas Gerais* (UFMG). In November 2020, the program was expanded to faculty, public servants and students from the UFMG, in Belo Horizonte, where the program was kept in operation until March 2023. Teófilo Otoni has a population of 142.030 inhabitants and a human development index (HDI) of 0.70; Divinópolis has a population of 248.581 inhabitants and an HDI of 0.76; Belo Horizonte has a population of 2.392.678 inhabitants and an HDI of 0.81 [11].

### The telemonitoring service

Telemonitoring teleconsultations were conducted majority by the students. In case the students needed help monitoring patients, they could request help form the nurses. The TeleCOVID-MG staff, including the medical students, was trained on the best scientific evidence available about COVID-19, following the recommendations from the World Health Organization (WHO) and the Brazilian Health Ministry guidelines [12–14].

The telemonitoring consultations were performed through phone calls. The calls were performed every 48 h for patients with no risk factors for severe COVID-19 and every 24 h for those with at least one risk factor. These risk factors included age ≥ 60 years-old, diabetes mellitus, chronic respiratory diseases, cardiovascular diseases, obesity, chronic kidney disease, immunosuppression, chromosomal diseases and high-risk pregnancy [13, 14].

To establish and maintain the link between the patient and the assistant, medical students promptly introduced themselves as soon as the patient answered the phone call. This introduction aimed to reduce communication noise and build trust. In each call, the students were instructed to ask the patients about their symptoms, the severity and progression of those symptoms, compared to the previous contact, and if they had any of the warning signs—hypotension, dyspnea, and high fever, especially if refractory. On the other hand, patients usually had doubts about the length of isolation; what to do when they had contact with a person with a confirmed diagnosis of covid; respiratory etiquette; specific places to test for COVID-19; when to seek in-person medical care.

Additionally, given the pandemic scenario, the students often provided psychological support, particularly for patients requiring daily telemonitoring.

Taking the patients’ answers into account and according to the guidelines provided, there were four possibilities after the monitoring teleconsultation: the students might refer the patients to the second or third levels for nursing or medical evaluation; the patient might stay in the service for further monitoring on the fourth level; the patient might be discharged; or the patient might be advised to seek onsite evaluation at a primary care center or an emergency department.

In situations when there was no response from the patient to the monitoring phone calls, the contact attempts were maintained for 48-72 h. After this period, if the student could not reach the patient, he was discharged from the TeleCOVID-MG program.

Patients were telemonitored for at least ten days from the onset of the symptoms, and then discharged if they were asymptomatic or had only residual symptoms in the previous 24 h. Patients who remained symptomatic beyond the tenth day were telemonitored at least until the fourteenth day from the symptoms’ onset. Whenever it was identified an alarm sign or any kind of complication during the clinical course of the illness, the medical students were able to refer the patient for a nursing or medical assessment at levels 2 or 3, or to advise the patient to seek for onsite evaluation at the primary care unit or at the emergency department.

The individuals assisted by the telemonitoring were then followed in another study, regarding the presence of post-covid-19 cognitive symptoms. [15]

The students participated in an online training that included instructions about how to operate the TeleCOVID-MG platform and how to carry out the clinical tasks of the telemonitoring level, as the correct identification of warning signs, such as respiratory and cardiovascular abnormalities—such as observing the frequency of coughing during the call, breathless speech, hoarseness and forced breathing -, lethargy, mental confusion, decompensation of underlying diseases, fever for more than three days or return of the fever after 48 h afebrile, and the correct identification of residual symptoms, such as post infectious cough. On the other hand, to maintain a link with the patient, as soon as the patient answered the phone call, medical students promptly introduced themselves, in order to reduce mistrust. Furthermore, given the pandemic scenario, in many cases the students served as psychological support, especially for patients who demanded daily telemonitoring. Finally, students were permanently supervised, and each doubt was immediately discussed with the supervising doctor/professor.

The students' contribution depended on their filiation, personal availability, contribution to other activities of the Telehealth Center, and epidemiological need of assistance. Usually, the undergraduates created schedules to better suit their availability, always maintaining two or more per shift, depending on epidemiological necessity. Shifts usually lasted between 3 to 6 h.

### Assessment of the students’ participation in the TeleCOVID-MG program

To assess the participation of the medical students in the telemonitoring service of the TeleCOVID-MG program, a questionnaire with 27 questions was developed (six dissertative and 21 multiple-choice questions). The questions addressed *the student’s profile*: sex, age, graduation year, university; *the system’s usability*: how many times the students used the system, the difficulties in contacting and assisting the patients, how they contacted the patients (own SIM card, private number) and *the satisfaction in participating in the TeleCOVID-MG program*, assessed based on Likert-scale questions about strengths and weakness, with answers including completely agree, partially agree, indifferent, partially disagree and completely disagree. The questionnaire was included on the Google Forms® platform and sent via email, from August to September, 2022, to all students who were part of the TeleCOVID-MG telemonitoring staff. As an exclusion criteria, there were undergraduates who used the system less than 5 times.

The medical students involved in the telemonitoring activities were mainly from four different medical schools, three public and one private: *Universidade Federal dos Vales do Jequitinhonha e Mucuri—campus Teófilo Otoni* (UFVJM), *Universidade Federal de São João Del Rei, campus Divinópolis* (UFSJ), UFMG and *Faculdade de Ciências Médicas de Minas Gerais* (FCMMG), respectively.

### Data analysis

Data were described by absolute and relative frequencies for counts, and median and interquartile range (IQR) for age, the only continuous variable, as it had a non-normal distribution. Data were processed and analyzed using the IBM SPSS Statistics for Windows software (Version 21.0. Armonk, NY: IBM Corp.).

## Results

From June 2020 to July 2022, the TeleCOVID-MG program performed 155,913 teleconsultations and attended to 39,647 people. The breakdown is as follows: at the nursing level, 38,292 consultations were conducted for 29,426 patients; at the medical level, 23,681 consultations for 16,341 patients; and for monitoring, 94,518 consultations for 32,522 patients. As patients could have had more than one consultation at each level and in different levels, the number of consultations is higher than the number of patients. Overall, 1,491 teleconsultations (1.0%) were conducted solely to clarify doubts, and 87,390 (56.1%) were monitoring teleconsultations.

Since the implementation of the program in May 2020, 130 medical students contributed to the telemonitoring service and 60 (46.2%) of them answered the questionnaire. Three of the undergraduates referred using the system less than 5 times and therefore were excluded from the analysis. The characteristics of the students who answered the questionnaire are described in Table 1.

**Table 1:** Participants’ sociodemographic characteristics and their contribution time to the project (*n* = 60)

Characteristics	n (%) or median [IQR]
**Median age (years)**	25 [24–26]
**Women**	40 (66.7%)
**Affiliation**	
Federal universities	55 (91.7%)
Private medical school	5 (8.3%)
**Graduation year**	
First	3 (5%)
Second	7 (11.7%)
Third	12 (20%)
Fourth	17 (28.3%)
Fifth	13 (21.7%)
Sixth	8 (13.3%)
**Contribution time to the project**	
Less than a month	1 (1.6%)
1 to 3 months	11 (18.3%)
3 to 6 months	10 (16.7%)
6 months to a year	16 (26.7%)
More than a year	22 (36.7%)
**Number of times using the system**	
5 to 10 times	3 (5.0%)
11 to 20 times	4 (6.7%)
More than 20 times	12 (20%)
More than 100 times	41 (68.3%)

*IQR* Interquartile Range

Regarding the usability and satisfaction with the system, among the positive perceptions, the students considered the system intuitive (100%), effective in the provision of quality care (100%), valuable for clinical practice (96.8%) and easy to use (98.4%). The only negative aspect pointed out was the eventual instability of the system, assessed by 46.0% of the undergraduates. Detailed data are shown in Table 2.

**Table 2:** Usability and satisfaction with the TeleCOVID-MG teleconsultation system by the medical students who participated in telemonitoring activities (*n* = 60)

	**Completely agree**	**Partially agree**	**Indifferent**	**Partially disagree**	**Completely disagree**
The TeleCOVID-MG teleconsultation system is intuitive	48 (80.0)	12 (20.0)	-	-	-
The TeleCOVID-MG teleconsultation system allows you to record all the relevant information about the patient	35 (58.3)	24 (40.0)	-	1 (1.7)	-
Following theTeleCOVID-MG teleconsultation system’s guidelines, I was able to provide quality patient care	47 (78.3)	13 (21.7)	-	-	-
The fields of the TeleCOVID-MG teleconsultation system are easy to fill in	43 (71.7)	16 (26.7)	1 (1.6)	-	-
The TeleCOVID-MG teleconsultation system is stable and errors do not occur during its use	3 (5.0)	29 (48.3)	4 (6.7)	19 (31.7)	5 (8.3)
I believe that the service can be useful in clinical practice, for the care of patients with suspected COVID-19	49 (81.6)	9 (15.0)	1 (1.7)	1 (1.7)	-
I felt satisfied with the use of TeleCOVID-MG teleconsultation system	48 (80.0)	11 (18.3)	1 (1.7)	-	-

On the other hand, regarding the contact with the patient, 95.0% pointed out difficulties in contacting the patient, even though only 23.4% reported calling with an unidentified number. This was a limitation that 55.0% of the students consider to have jeopardized the service’s efficiency somehow. In addition, 60.0% of the undergraduate students believed that patients have been insecure in some instances with the approach of medical students. Finally, 69.8% of the undergraduates experienced technical difficulties during care. However, 96.6% of them affirmed that they were promptly solved, mainly by consulting the program coordinator, the medical advisor or the protocol developed by the TNMG. In view of the pros and cons of the system, 98.3% of the undergraduates reported being satisfied with their performance and would recommend the service to an acquaintance.

## Discussion

Medical students played a pivotal role in the success of the TeleCOVID-MG, actively engaging in remote monitoring and care delivery initiatives. In our experience, students used the TeleCOVID-MG system numerous times, which not only demonstrates a profile of high productivity and commitment, but also reinforces the importance of medical undergraduates in the teleassistance program. This is confirmed by the 87,390 telemonitoring calls provided by level 4, which is equivalent to 56.1% of total attendances during the program period.

In this context, it is important to highlight that the TeleCOVID-MG’s main purpose was to minimize the overload for the public and private healthcare systems by providing care that could be delivered whilst the patient was at home. Therefore, the proactive involvement of medical students expanded the reach of the program to underserved populations and remote communities, potentially having contributed to public health outcomes. Their participation allowed high capillarity and provision of care to patients that probably would not be assisted in the pandemic context, in which several health services and public transportation worked with restricted capacity, especially affecting low-income regions with expressive rural populations. By conducting telemonitoring calls and providing patient care, medical students demonstrated a strong commitment to addressing health inequalities and ensuring equitable access to healthcare services. In addition to that, health professionals’ overload was mitigated by the volume of telemonitoring consultations performed by medical undergraduates in the emergence context, as several students could be supervised by a single doctor, whose assistance could be reserved to cases with risk factors or alarm signs.

Although healthcare access in remote communities is still a challenge for the effective implementation of telemedicine, the TeleCOVID-MG made it possible for the population to be monitored during the COVID-19 pandemic. Due to challenges in digital health accessibility and internet connectivity, the use of telephone-based teleconsultations as an alternative mode of communication was of utmost importance to meet the needs of vulnerable populations. The Regional Center for Studies on the Development of the Information Society, in 2020, pointed out that 93% of the population with 10 or more years had a cell phone, and there was a significant increase regarding rural areas’ users: from 79% in 2019 to 91% in 2020 [16]. However, access to internet connection in rural areas and remote site is still impaired. Therefore, there are satisfactory prospects concerning digital accessibility and the use of telemedicine in Brazil, with a projection that 100% of the population will be online in 2028, if the growth rate continues [17].

By actively participating in the TeleCOVID-MG program, medical students not only gained valuable clinical experience but also contributed to their professional development and understanding of public health principles. The uncertainties brought by COVID-19 to the educational system promoted the development and implementation of remote learning worldwide to ensure students' safety while allowing them to continue their academic formation. Many health sciences undergraduate courses continued with online teaching only, even for subjects with a higher teaching load originally focused on practical activities, which might have jeopardized the quality of learning for their students [18–20]. However, many medical students were not included in virtual classes due to the higher proportion of practical classes, especially in the last two years of medical school.

In this context, the participation in the TeleCOVID-MG monitoring activities described in this study became a useful tool in the educational field by enabling the continuity of medical practice and care through telemedicine. This allowed students to improve skills such as communication, doctor-patient relationships, clinical reasoning, decision-making, and recording information in electronic medical records on the program's platform. The students' experiences were largely positive, with most strongly agreeing that the TeleCOVID-MG service provided good quality assistance to a population that might not have been assisted in the pandemic context, while their performance in telemonitoring contributed to their medical education.

Additionally, their dedication and commitment to serving their communities underscored the critical role of medical education in preparing future healthcare professionals to respond effectively to global health challenges. Similar experiences reported by other authors during the pandemic have highlighted the benefits of such adaptations in medical education, including improved communication skills, increased empathy, and better patient management. They also perceive the experience as valuable, enhancing their knowledge, and recognizing the important role telemedicine will play in their future careers [21–23]. Integrating these findings with our experience further reinforces the value of telemedicine and remote learning in medical training during unprecedented times.

Other successful experiences have been reported in the literature. A recent study described the experience of 12 undergraduate medical students participating in an inpatient teleconsultation program, remotely assisting attending consultants in the infectious diseases and nephrology divisions, including COVID-19 patients. Satisfaction surveys from the participating students and faculty were largely positive, with most agreeing or strongly agreeing on the importance of teaching medical students about telehealth. The authors highlighted that this modality of care provided a satisfactory educational gain for the medical students who were unable to carry out their practical activities during the COVID-19 pandemic [24]. Another study reported the experience of 64 fourth-year medical students on a four-week elective service during the suspension of clinical rotations. Satisfaction surveys indicated that the medical undergraduates felt well-prepared for patient encounters and appreciated the opportunity to develop important skills, particularly with telehealth technologies. The authors emphasized a considerable alleviation of the healthcare burden with this experience, given the volume of appointments handled by the students—over 1400 teleconsultations [19].

The increased use of health technologies not only allowed the continuity of patient care in a time of global crises, decreasing the in-person consultations overload, but also contributed to prevent the possible delay in the student’s graduation by allowing the continuity of care practice. Thus, these technologies minimized the possible future prejudice for the community [25], although the intention of telemedicine in medical training is not to replace face-to-face clinical practice, but to add new perspectives regarding health services [26]. The participation of undergraduate medical students in this emerging tool at a delicate moment in national health is essential to expand concepts, strategies and overcome paradigms, as telemedicine is now a reality. In this context, the motivation for continuing learning, the permanent pursuit for latest health guidelines and protocols, as well as the constant need for training and discussions with other health professionals about their own experiences made it possible to broaden the possibilities of different health assistance modalities. Furthermore, students were able to use techniques for collecting information from patients over the telephone, using the subtleties of slurred speech or flu-like symptoms (coughing, sneezing, hoarseness), allowing not only to hear what the patient was complaining about, but to ask questions active about some noticeable symptom, but not mentioned in the call. Therefore, to assess the accomplishment of curricular necessities, a recent study established objectives to the medical students: (1) understanding patients with flu signs and symptoms remotely, (2) developing fluency with telehealth technologies to support virtual consultations, (3) learning with health services professionals and implement techniques to interact with patients during virtual consultations, (4) use telehealth modalities to assess clinical status and stratify into risk and severity levels, (5) develop a plan of care, follow-up and counseling of patients during virtual consultations [19]. These parameters can be used to assess other telehealth programs that count with undergraduates’ participation.

It is important to recognize that the incorporation of telehealth is not exempt from challenges and potential disadvantages. Studies have shown that among the main limitations pointed out by students who participated in telemedicine programs during the pandemic context are the possibility of reduced face-to-face interaction among students, patients, and the healthcare team, the impossibility to perform physical examinations, and challenges in addressing mental health issues when the patient did not have a quiet place to discuss sensitive matters [27, 28] Thus, the lack of exposure of students to in-person clinical situations may result in some educational loss. [28] Therefore, while telehealth programs offer undeniable benefits in terms of enhancing and developing teaching methodologies, it is essential to critically consider their limitations and seek strategies to mitigate potential negative impacts on the process of clinical teaching and learning.

The analysis of the questionnaire made it possible to establish the main strengths and weaknesses of the system, as well as the profile of medical undergraduates who participated in the TeleCOVID-MG project. In this context, it was observed that students were, overall, in more advanced periods of the course, and that 65% of them have used the TeleCOVID-MG system more than 100 times. Among the challenges, the difficulty in contacting patients and possible system instabilities were the most reported. Since the TeleCOVID-MG platform was under constant construction, the need for system maintenance promotes momentary interruptions in the telemonitoring, increasing the waiting list. In addition, the overload of internet providers in the pandemic context, along with the increase in home office and online academic activities, contributed to the unstable environment [29]. Many of the students working in the program, due to the suspended face-to-face activities, returned to their home cities, where the internet supply was sometimes limited, which may also contribute to the technical difficulties.

### Limitations

The limitations of this study are related to the difficulty of locating all the students who worked in the telemonitoring activities. However, we believe that the sample was representative, and to the best of our knowledge this study reported the highest numbers of patients being telemonitored since the beginning of COVID-19 pandemic.

### Future directions

The active participation of medical students in telemedicine programs such as TeleCOVID-MG holds profound significance for the future of healthcare delivery. Not only do medical students play a crucial role in delivering care to underserved populations and remote communities, but they also contribute to shaping the evolution of telemedicine itself. By engaging in telemonitoring consultations and advocating for the integration of telehealth technologies into medical education curricula, medical students drive innovation and promote the adoption of digital solutions in healthcare. Their proactive involvement underscores the importance of providing medical students with practical experiences in telemedicine to prepare them for future healthcare challenges. As advocates for equitable access to healthcare and technological advancement, medical students are poised to lead the way in transforming healthcare delivery and addressing the evolving needs of patients and communities.

There are other potential future directions to be considered:

- Long-term impact assessment: further studies may assess the long-term impact of participation in the TeleCOVID-MG program on the medical students' clinical skills, career choices, and attitudes towards telemedicine. Additionally, long-term patient outcomes should be assessed. Specifically related to this program, a group of undergraduate students participated in a long COVID assessment, calling patients 12 months after acute COVID-19 to inquire about persistent symptoms. In addition to specific publications on long COVID assessment, they worked on educational material published by the Pan American Health Organization to inform the population about long COVID-19.
- Comparison with traditional clinical rotations: further studies should compare the effectiveness of telemonitoring experiences, such as those offered by the TeleCOVID-MG program, with traditional in-person clinical rotations in terms of educational outcomes, patient outcomes, and cost-effectiveness.
- Patient satisfaction and outcomes: further studies should explore patient satisfaction and clinical outcomes associated with telemonitoring services provided by medical students with the support of medical specialists compared to those provided by experienced healthcare professionals only, as well as to assess how the participation of undergraduate students may contribute to reducing healthcare disparities and improving access to care, particularly for underserved populations and those in rural or remote areas.
- Enhancement of telehealth platforms: it is important to work on enhancing the telemonitoring platforms. The feedback from medical students and other users are useful to improve usability, stability, and effectiveness in delivering telehealth services.
- Expansion to other healthcare settings: to assess the feasibility and effectiveness of implementing similar telemonitoring programs in other healthcare settings, such as rural clinics, primary care centers, and specialty hospitals.
- Interprofessional collaboration: it is important to explore opportunities for interprofessional collaboration in telehealth settings by involving students from other healthcare disciplines, such as nursing, pharmacy, and social work, to provide comprehensive care to patients.

## Conclusions

This study reports a successful experience of undergraduate medical students in a COVID-19 telemonitoring program. The TeleCOVID-MG program enabled the continuity of clinical practice in a context of suspension of classes, the learning of emerging healthcare tools and the development of new abilities in the medical-patient relationship. Overall, the medical students were satisfied with their participation and they had an important role in the assistance of patients from low-income regions.

### Supplementary Information

Supplementary Material 1.

## Data Availability

The datasets used and/or analyzed during the current study are available from the corresponding author on reasonable request.

## Abbreviations

FCMMG: Faculdade de Ciências Médicas de Minas Gerais
HDI: Human Development Index
IQR: Interquartile Range
TNMG: Telehealth Network of Minas Gerais
UFMG: Universidade Federal de Minas Gerais
UFSJ: Universidade Federal de São João Del Rei
UFVJM: Universidade Federal dos Vales do Jequitinhonha e Mucuri
WHO: World Health Organization
