# Supplementary material for: Medical students experience in working in a public COVID-19 telehealth program: a descriptive study
Source: BMC Med Educ. 2024 Jul 12;24:756. doi: 10.1186/s12909-024-05722-6 (PMC11245774; doi:10.1186/s12909-024-05722-6)
Supplement: Supplementary file 1 — Supplementary Material 1. [file 12909_2024_5722_MOESM1_ESM.pdf]

Questionário de avaliação Telecovid-MG: Usabilidade e satisfação de acadêmicos no sistema de telemonitoramento Telecovid-MG (Teófilo Otoni, Divinópolis e Monitora UFMG)

Prezados acadêmicos,

Queremos avaliar sua experiência como colaboradores do projeto Telecovid-MG. Para isso, elaboramos um questionário de usabilidade e satisfação apresentado abaixo. Sua opinião é muito importante para melhorarmos o serviço.

Caracterização do acadêmico

1. Qual o seu e-mail?

2. Qual o seu sexo?

   Feminino

   Masculino

3. Qual sua idade?

4. Em que ano você iniciou o curso de Medicina?

5. Em qual Universidade?

   UFMG

   UFSJ

   UFVJM

   Outro:

6. Qual período você estava cursando quando iniciou as atividades no projeto?

   5o período

   6o período

   7o período

   8o período

   9o período

   10o período

   11o período

   12o período

   Outro:

7. Qual o seu período de contribuição no projeto?

   Menos de 1 mês

   1 a 3 meses

3 a 6 meses

6 meses a 1 ano

Mais de 1 ano

8. Quantas vezes você utilizou o sistema até o momento?

Menos de 5 vezes

5 a 10 vezes

11 a 20 vezes

Mais de 20 vezes

Mais de 100 vezes

Experiência com a plataforma

Marque se você concorda ou discorda de cada afirmação.

9. As telas do sistema de teleconsultas Telecovid-MG são de fácil entendimento.

Concordo totalmente

Concordo parcialmente

Indiferente

Discordo parcialmente

Discordo totalmente

10. O sistema de teleconsultas Telecovid-MG permite registrar todas as informações relevantes sobre o atendimento do paciente.

Concordo totalmente

Concordo parcialmente

Indiferente

Discordo parcialmente

Discordo totalmente

11. Seguindo as telas do sistema de teleconsultas Telecovid-MG, fui capaz de fazer um atendimento de qualidade ao paciente.

Concordo totalmente

Concordo parcialmente

Indiferente

Discordo parcialmente

Discordo totalmente

12. Os campos do sistema de teleconsultas Telecovid-MG são de fácil preenchimento.

Concordo totalmente

Concordo parcialmente

Indiferente

Discordo parcialmente

Discordo totalmente

13. O sistema de teleconsultas Telecovid-MG é estável e não ocorrem erros durante seu uso.

Concordo totalmente

Concordo parcialmente

Indiferente

Discordo parcialmente

Discordo totalmente

14. Eu acredito que o serviço pode ser útil na prática clínica, para o atendimento dos pacientes com suspeita de Covid-19

Concordo totalmente

Concordo parcialmente

Indiferente

Discordo parcialmente

Discordo totalmente

15. Eu me senti satisfeito com o uso do sistema de teleconsultas Telecovid-MG.

Concordo totalmente

Concordo parcialmente

Indiferente

Discordo parcialmente

Discordo totalmente

16. Eu recomendaria o serviço a um conhecido

Concordo totalmente

Concordo parcialmente

Indiferente

Discordo parcialmente

Discordo totalmente

Particularidades da experiência como acadêmico

17. Você usou, majoritariamente, número de telefone/chip próprio ou o(s) disponibilizado(s) pelo projeto?

Número de telefone/Chip próprio

Número de telefone/Chip disponibilizado pelo projeto

18. Você realizou as ligações em modo privado ou sem identificação do número de telefone?

Sim

Não

19. Você teve dificuldades técnicas durante o telemonitoramento?*

Sim

Não

20. Se sim, como essas dificuldades foram sanadas?

Pesquisa no protocolo

Pesquisa na literatura

Perguntei ao coordenador

Perguntei ao professor

Perguntei a outro profissional

Não foram sanadas

Outro:

21. Você teve dificuldades de contato com os pacientes?*

Sim, sempre

Sim, às vezes

Sim, raramente

Não

22. Se sim, você acha que isso prejudicou a efetividade do serviço?

Sim

Não

23. observou alguma insegurança dos pacientes por serem contactados por acadêmicos?*

Sim, sempre

Sim, às vezes

Sim, raramente

Não

24. Você ficou satisfeito com sua contribuição como acadêmico no serviço?*

Sim, totalmente satisfeito

Sim, parcialmente satisfeito

Não fiquei satisfeito ou insatisfeito

Fiquei insatisfeito

Considerações finais

25. Vantagens/pontos fortes da sua participação como acadêmico no serviço Telecovid-MG.

26. Desvantagens/pontos fracos da sua participação como acadêmico no serviço Telecovid-MG.

27. Deseja fazer algum comentário adicional sobre o sistema ou o serviço?
